# Life Supported by Injections

**Published:** 1874-02

**Authors:** 


LENGTH OF TIME LIFE MAY BE SUPPORTED BY INJECTIONS.—Dr. Runze has reported a case in which the patient could not, for fifty-nine days, swallow any food (liquid or solid), and was nourished by means of injections containing the yolks of eggs, broths, soups, etc. During the first week, the patient became weak and complained of a painful sensation of thirst; the third week the patient did not experience any hunger, thirst or pain. There were but slight evacuations. The eighth week, however, an inflammation of the large intestine was set up, and death took place during the ninth week.

